# Contrast-Enhanced FLAIR (Fluid-Attenuated Inversion Recovery) for Evaluating Mild Traumatic Brain Injury

**DOI:** 10.1371/journal.pone.0102229

**Published:** 2014-07-16

**Authors:** Soo Chin Kim, Sun-Won Park, Inseon Ryoo, Seung Chai Jung, Tae Jin Yun, Seung Hong Choi, Ji-hoon Kim, Chul-Ho Sohn

**Affiliations:** 1 Department of Radiology, Seoul Metropolitan Government - Seoul National University Boramae medical center, Seoul, Korea; 2 Department of Radiology, Seoul National University College of Medicine, Seoul, Korea; 3 Department of Radiology, Korea University Guro Hospital, Seoul, Korea; 4 Department of Radiology, Asan Medical Center, Seoul, Korea; 5 Department of Radiology, Seoul National University Hospital, Seoul, Korea; University of Wuerzburg, Germany

## Abstract

**Purpose:**

To evaluate whether adding a contrast-enhanced fluid-attenuated inversion recovery (FLAIR) sequence to routine magnetic resonance imaging (MRI) can detect additional abnormalities in the brains of symptomatic patients with mild traumatic brain injury.

**Materials and Methods:**

Fifty-four patients with persistent symptoms following mild closed head injury were included in our retrospective study (M∶F = 32∶22, mean age: 59.8±16.4, age range: 26–84 years). All MRI examinations were obtained within 14 days after head trauma (mean: 3.2±4.1 days, range: 0.2–14 days). Two neuroradiologists recorded (1) the presence of traumatic brain lesions on MR images with and without contrast-enhanced FLAIR images and (2) the pattern and location of meningeal enhancement depicted on contrast-enhanced FLAIR images. The number of additional traumatic brain lesions diagnosed with contrast-enhanced FLAIR was recorded. Correlations between meningeal enhancement and clinical findings were also evaluated.

**Results:**

Traumatic brain lesions were detected on routine image sequences in 25 patients. Three additional cases of brain abnormality were detected with the contrast-enhanced FLAIR images. Meningeal enhancement was identified on contrast-enhanced FLAIR images in 9 cases while the other routine image sequences showed no findings of traumatic brain injury. Overall, the additional contrast-enhanced FLAIR images revealed more extensive abnormalities than routine imaging in 37 cases (p<0.001). In multivariate logistic regression analysis, subdural hematoma and posttraumatic loss of consciousness showed a significant association with meningeal enhancement on contrast-enhanced FLAIR images, with odds ratios 13.068 (95% confidence interval 2.037 to 83.852), and 15.487 (95% confidence interval 2.545 to 94.228), respectively.

**Conclusion:**

Meningeal enhancement on contrast-enhanced FLAIR images can help detect traumatic brain lesions as well as additional abnormalities not identified on routine unenhanced MRI. Therefore contrast-enhanced FLAIR MR imaging is recommended when a contrast MR study is indicated in a patient with a symptomatic prior closed mild head injury.

## Introduction

Traumatic brain injury (TBI) often leads to neurocognitive deficits and neurobehavioral abnormalities. Even with mild traumatic brain injury, many patients have long-term neuro-logic or neuropsychologic abnormalities [1], [2]. As a result, imaging evaluation for detection of traumatic lesions has drawn much attention. Many prior studies have reported that magnetic resonance imaging (MRI) can reveal traumatic lesions responsible for clinical symptoms and signs in patients with negative computed tomography (CT) examinations [3]. Currently, susceptibility-weighted imaging (SWI) is more sensitive than conventional MR imaging for the detection of microhemorrhages [4], [5]. Diffusion tensor imaging (DTI) has emerged as a valuable additional technique to evaluate traumatic brain abnormalities [6]–[10]. However, these specialized advanced sequences require long imaging time leading to an increased incidence of movement artifacts [11], [12], and requirement of special imaging hardware.

The fluid-attenuated inversion recovery (FLAIR) is a special inversion recovery sequence using a long repetition time (TR) and echo time (TE) and an inversion time that effectively suppresses signals from free water in cerebrospinal fluid (CSF), thus allowing to highlight hyperintense lesions adjacent to CSF containing spaces. Although FLAIR images are heavily T2-weighted images, these MR images also have mild T1-weighting, which is responsible for contrast enhancement. Because of the combination of T2 prolongation, the usual mechanism for hyperintense lesion on FLAIR images, and T1 shortening from contrast material with CSF signal suppression, contrast-enhanced FLAIR MR imaging is highly sensitive to the detection of subtle cortical abnormalities such as meningeal infection, inflammation and metastases [13]–[15]. Goo et al. reported the case demonstrating abnormal meningeal enhancement in patient with a subdural hematoma on contrast-enhanced FLAIR MR image [16]. However, to our knowledge, the clinical importance of this sequence in patients with head trauma has not been evaluated thus far.

Therefore, the purpose of the present study was to evaluate whether contrast-enhanced FLAIR MR imaging can help detect abnormalities in the brains of symptomatic subjects after mild traumatic brain injury and to determine the usefulness of additional contrast-enhanced FLAIR MR imaging.

## Materials and Methods

This retrospective study was approved by the institutional review board of Seoul Metropolitan Government - Seoul National University Boramae medical center, and patients' informed consent was waived.

### Patient Population

By using a computerized search of our hospital's medical records from October 2010 to February 2012, we identified 91 consecutive patients who visited our emergency department and had undergone brain MR examination because of persistent symptoms following mild closed head injury. Inclusion criteria for the mild traumatic brain injury were based on the American Congress of Rehabilitation Medicine [Glasgow Coma Score of 13–15, post-traumatic loss of consciousness (LOC) (if present) <30 min, post-traumatic amnesia (PTA) as measured by the Galveston orientation and amnesia test (if present) <24 h]. Nineteen patients with non-contrast-enhanced MR imaging were excluded. Seven patients with no available medical record about clinical background information were also excluded. Five patients were excluded because they had history of brain surgery or prior head injury before the traumatic event. Six patients were excluded because there was an interval of >14 days between the date of their injuries and MR examinations. A total of 54 patients were included in our retrospective study (M∶F = 32∶22, mean age: 59.8±16.4, age range: 26–84 years). The reasons for head injury were a fall in 33 patients, traffic accident in 17 patients, violence in 4 patients. CT was performed in all patients during acute hospitalization. All CT examinations found no abnormalities. All MRI examinations were obtained within 14 days after head trauma (mean: 3.2±4.1 days, range: 0.2–14 days). The summary of demographic and clinical characteristics of patients is shown in Table 1.

**Table 1:** The summary of demographic and clinical characteristics of patients.

Variable	Value
**Patients (n)**	54
**Age (years)**	
Mean	59.8 (±16.4)
Median (range)	63
**Sex (women/men)**	22/32
**Underlying disease (n)**	
No	34
Yes	20
Diabetes mellitus	8
Hypertension	7
Alzheimer's disease	3
Past history of stroke	2
**Time interval (days)**	
0–3	37
4–7	10
8–14	7
**Reason**	
Fall	33
Traffic accident	17
Violence	4
**GCS**	
13	3
14	3
15	48
**Post-traumatic amnesia (n)**	12
**Post-traumatic LOC (n)**	24

Note − GCS indicates Glasgow Coma Score; LOC, loss of consciousness.

### MR Imaging Technique

All studies were performed with 3 T MR imaging system (Achieva; Philips Medical Systems, Best, Netherlands) using a 16-channel head coil. Following the acquisition of sagittal scout T1-weighted images, spine-echo T2-weighted (TR/TEeff 3000/100 ms), spine-echo T1-weighted (TR/TE 260/10 ms; FA 70°), GRE T2*-weighted (TR/TE 724/16 ms; flip angle 18), and T2-FLAIR (TR/TEeff/TI 11000/120/2800 ms) were obtained. After the intravenous administration of 0.1 mmol/kg body weight gadobutrol (Gadovist, Bayer Schering Pharma, Berlin, Germany) over 1 minute, spine-echo T1-weighted and T2-FLAIR images were obtained. The acquisition time for contrast-enhanced FLAIR MR images was 2 minutes 40 seconds to 3 minutes 10 seconds (identical to non-contrast-enhanced FLAIR MR). All images were acquired with a section thickness of 5 mm, an intersection gap of 2 mm, a field of view of 18×18–22×22 cm, and a matrix of 256×192.

### Image analysis

Two experienced neuroradiologists (K.S.C. and R.Y.S. with 6 years of experience) reviewed the anonymized brain images by the consensus method. At the first session, they evaluated 50% of the cases using 2D T1, T2, FLAIR, GRE, and contrast-enhanced T1-weighted images as a routine image sequence and the other 50% using the combination of routine images and contrast-enhanced FLAIR images. After a 4-week interval, the cases were switched and read again. Each case was assessed for the presence or absence of traumatic brain lesions. Then each lesion was further described as follows: epidural hematoma (EH), subdural hematoma (SDH), subarachnoid hemorrhage (SAH)/intraventricular hemorrhage (IVH), brain contusion/intraparenchymal hemorrhage, and diffuse axonal injury (DAI). In addition, reviewers recorded the presence or absence and location of meningeal enhancement on the contrast-enhanced FLAIR images, dividing them into 3 categories: diffuse meningeal enhancement along both cerebral convexities; localized enhancement over part of 1 cerebral convexity; and enhancement along the falx only. In the localized meningeal convexity enhancement group, the location of meningeal enhancement was matched against the site of injury from medical records and/or scalp hematoma or laceration site demonstrated on imaging.

### Statistical Analysis

All statistical analyses were performed using the SPSS statistical software program (version 20.0, Chicago, III, USA). Comparison of the number of patients with traumatic brain lesions demonstrated on routine images and on the combination of routine images and contrast-enhanced FLAIR image was analyzed using the McNemar test. The unpaired student's *t*-test was used to determine whether the age and time interval of the group that showed meningeal enhancement on contrast-enhanced FLAIR images differed significantly from those of the group showing negative findings. Associations between the clinical values and meningeal enhancement on contrast-enhanced FLAIR were also evaluated by using logistic regression analysis. The data for each parameter were assessed for normality with the Kolmogorov-Smirnov test. In all tests, *P* values less than 0.05 were considered statistically significant.

## Results

**Figure 1:**
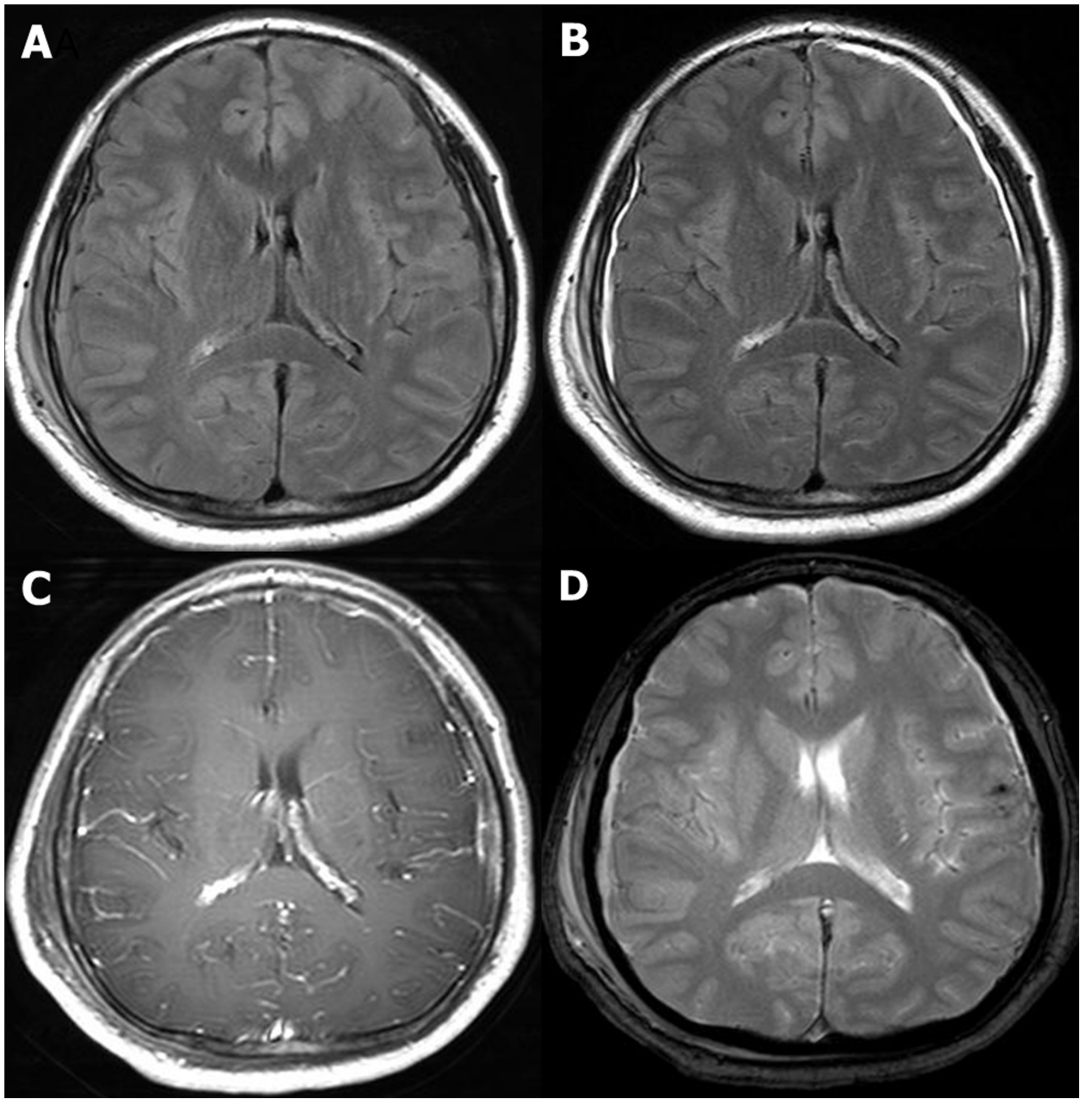
A 67-year- old male patient with a history of fall down 4 hours ago. Initial GCS score was 13. The duration of posttraumatic amnesia was 1(A) Unenhanced FLAIR MR image shows small amount of subdural hemorrhage with iso-signal intensity in Rt. parietal convexity. (B) contrast-enhanced FLAIR MR image clearly demonstrates meningeal enhancement along not only right convexity but also Lt. side. (C) contrast-enhanced T1 weighted image shows no definite enhancement. (D) GRE image depicts hemosiderin deposition only in Lt. cerebral cortex.

**Figure 2:**
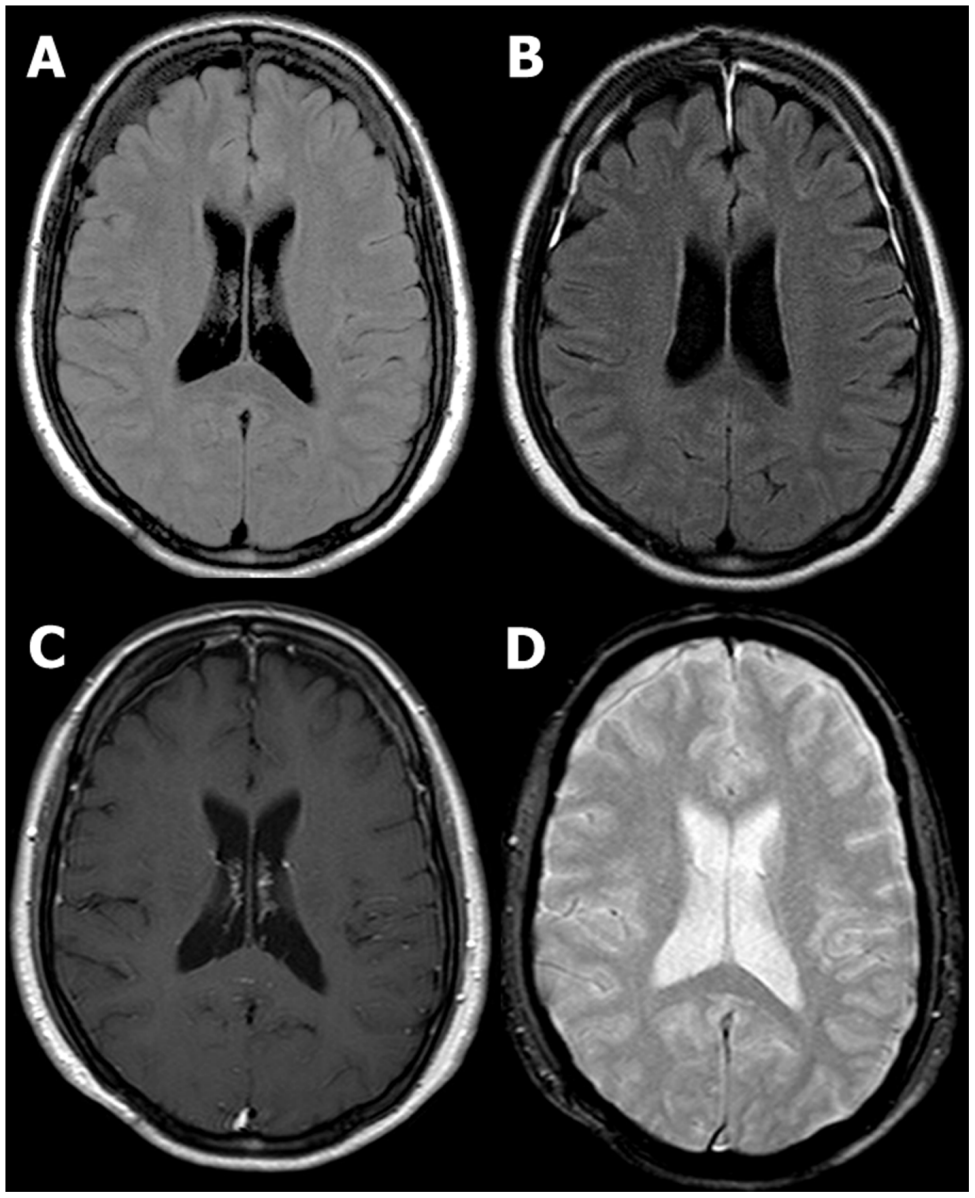
A 60-year- old female patient with a history of assault 10 hours ago. Initial GCS score was 15. The duration of posttraumatic amnesia was 3(B) Contrast-enhanced FLAIR MR image helped to detect small amount of subdural hemorrhage in both frontal convexity which was initially missed, after reviewing meningeal enhancement. No demonstrable abnormality was found on unenhanced FLAIR (A), contrast-enhanced T1 weighted (C) and GRE (D) MR images.

**Figure 3:**
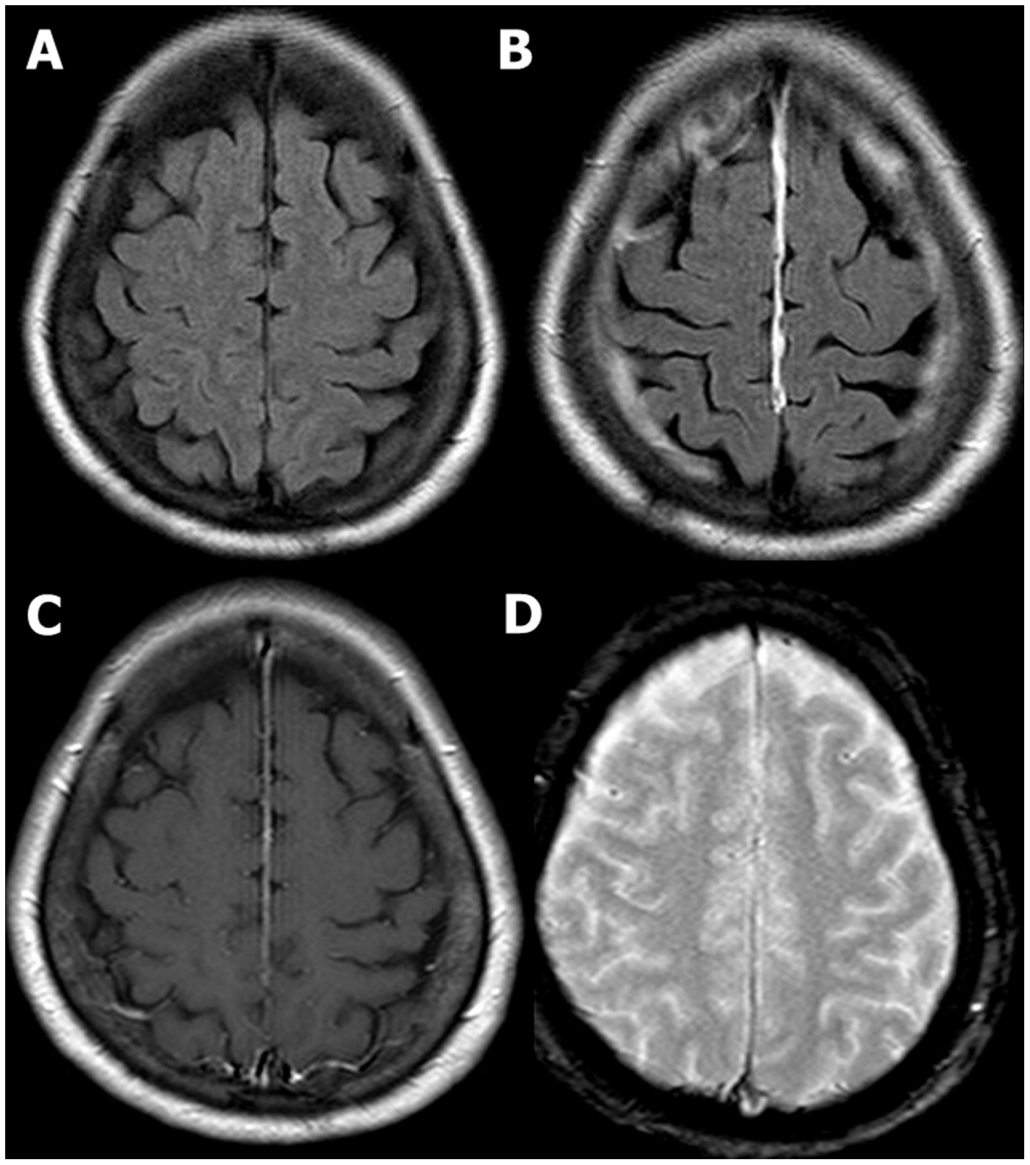
A 63-year- old female patient with a history of fall down 2 days ago. Initial GCS score was 15. She had transient episode of loss of consciousness less than 30(B) Only contrast-enhanced FLAIR MR image reveals abnormal finding – meningeal enhancement along falx. No demonstrable abnormality was found on unenhanced FLAIR (A), contrast-enhanced T1 weighted (C) and GRE (D) MR images.

**Figure 4:**
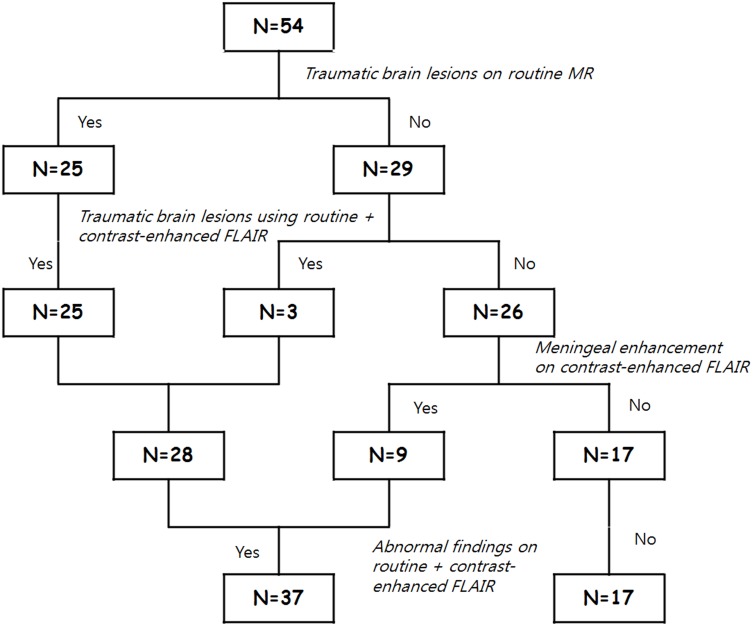
Diagram of the study results.

Twenty-five patients with traumatic brain lesions were identified among 54 total patients after reviewing routine MRI imaging sequences. Out of the 25 patients, 22 patients showed SDH, 13 patients had SAH/IVH, 9 patients demonstrated brain contusion/intraparenchymal hemorrhage, and 3 patients had DAI. Meanwhile, 28 patients with traumatic brain lesions were detected on routine image sequence plus contrast-enhanced FLAIR (Figure 1, 2). Two patients with minimal amounts of SDH and 1 patient with SAH were additionally detected on routine image sequences in retrospect after the findings were depicted on contrast-enhanced FLAIR images (p = 0.25). In 9 patients, only contrast-enhanced FLAIR images demonstrated abnormal meningeal enhancement confined to the falx while the other routine image sequences showed no findings of traumatic brain injury (Figure 3). Overall, the combination sequence detected 37 patients with abnormalities, significantly more than on routine images alone (p<0.001) (Table 2, Figure 4).

**Table 2:** Comparison of the number of patients with traumatic brain lesions demonstrated on routine images and on the combination of routine images and contrast-enhanced fluid-attenuated inversion recovery (FLAIR) image.

	T2+T1+GRE+FLAIR+	Plus contrast-enhanced FLAIR
	Contrast-enhanced T1WI	
**1) Patient with TBI (n/54)** *	**25**	**28**
EH	0	0
SAH/IVH	13	14
SDH	22	24
Brain contusion/intraparenchymal hemorrhage	9	9
DAI	3	3
**p = 0.250**		
**1) + Patient with only meningeal enhancement on contrast-enhanced FLAIR (n/54)** *	**25+0**	**28+9**
**p<0.001**		

*Number of patients with traumatic brain lesions among a total of 54 patients.

Note − TBI indicates traumatic brain injury; EH, epidural hemorrhage; SAH, subarachnoid hemorrhage; IVH, intraventricular hemorrhage; SDH, subdural hemorrhage; DAI, diffuse axonal injury.

A total of 32 patients demonstrated meningeal enhancement on contrast-enhanced FLAIR images. With regard to the location of meningeal enhancement, 12 patients showed diffuse meningeal enhancement, 8 patients had localized convexity meningeal enhancement, and 12 patients showed meningeal enhancement only along the falx (Table 3). Out of the 8 patients who showed localized convexity meningeal enhancement, the enhancing meningeal site was opposite the injured area in 6 patients (contrecoup). In 1 patient, the site of meningeal enhancement was not obviously related to the area of injury. The exact site of injury could not be located in other patients.

**Table 3:** The location of meningeal enhancement on contrast-enhanced fluid-attenuated inversion recovery (FLAIR) image and the number of patients with traumatic brain lesions.

Factor	Positive meningeal enhancement				Negative*(n = 22/54)
	Diffuse (n = 12/54)	Localized (n = 8/54)	Falx cerebri (n = 12/54)	Total (n = 32/54)	
**TBI (n/28)** †	12	8	3	23	5
SAH/IVH	7	5	0	12	2
SDH	10	7	2	19	5
Brain contusion/intraparenchymal hemorrhage	6	1	0	7	2
DAI	0	1	1	2	1
**TI (days)** ‡	4.3±4.3	0.9±1.4	2.9±5.3	2.9±4.4	3.5±3.9

*Number of patients who showed no meningeal enhancement on contrast-enhanced FLAIR images among a total of 54 patients.

† Number of patients out of 28 patients who were identified as having a TBI using the combination of routine images and contrast-enhanced FLAIR image.

‡ The data are the means ± standard deviations.

Note − TBI indicates traumatic brain injury; SAH, subarachnoid hemorrhage; IVH, intraventricular hemorrhage; SDH, subdural hemorrhage; DAI, diffuse axonal injury; TI, time interval.

Table 4 shows the results from logistic regression analyses. At univariate analysis, the following clinical entities showed a significant association with meningeal enhancement on contrast-enhanced FLAIR images: SAH/IVH, SDH, posttraumatic LOC, and PTA. In multivariate logistic regression analysis, SDH, and posttraumatic LOC still remained significant, with odds ratios 13.068 (95% confidence interval 2.037 to 83.852), and 15.487 (95% confidence interval 2.545 to 94.228), respectively. In subgroup analysis which was used to evaluate the association between the subgroup showing enhancement confined to the falx and the group without any meningeal enhancement, univariate analyses revealed that SDH, posttraumatic LOC and PTA were related to the meningeal enhancement (Table 5). Multivariate analysis showed that SDH and LOC were significantly correlated with the enhancement, with odds ratios 6.878 (95% confidence interval 1.084 to 43.633), and 10.545 (95% confidence interval 1.973 to 56.375), respectively (Table 5).

**Table 4:** Association between meningeal enhancement on contrast-enhanced fluid-attenuated inversion recovery (FLAIR) images and clinical values.

Variable	Univariate Analysis		Multivariate Analysis	
	Odds Ratio	PValue	Odds Ratio	PValue
SAH/IVH	**6.000 (1.187, 30.324)**	**0.030**	2.356 (0.278, 19.981)	0.432
SDH	**4.969 (1.465, 16.856)**	**0.010**	**13.068 (2.037, 83.852)**	**0.007**
Brain contusion/intraparenchymal hemorrhage	2.800 (0.523, 14.992)	0.229	…	…
DAI	1.4 (0.119, 16.459)	0.789	…	…
Posttraumatic LOC	**11.217 (2.753, 45.697)**	**0.001**	**15.487 (2.545, 94.228)**	**0.003**
PTA	**9.545 (1.122, 81.198)**	**0.039**	12.640 (0.925,172.788)	0.057
Age*	…	0.558	…	…
TI*	…	0.651	…	…
GCS		0.716	…	…
GCS (15)	1		…	…
GCS (14)	0.000	0.999	…	…
GCS (13)	0.357 (0.030, 4.214)	0.414	…	…

*Determined with the unpaired student's *t*-test.

Note − SAH indicates subarachnoid hemorrhage; IVH, intraventricular hemorrhage; SDH, subdural hemorrhage; DAI, diffuse axonal injury; LOC, loss of consciousness; PTA, post-traumatic amnesia; TI, time interval; GCS, Glasgow Coma Score.

**Table 5:** Association between meningeal enhancement confined to the falx on contrast-enhanced fluid-attenuated inversion recovery (FLAIR) images and clinical values.

Variable	Univariate Analysis		Multivariate Analysis	
	Odds Ratio	PValue	Odds Ratio	PValue
SAH/IVH	0.000	0.998	…	…
SDH	**5.500 (1.073, 28.198)**	**0.041**	**6.878 (1.084, 43.633)**	**0.007**
Brain contusion/intraparenchymal hemorrhage	0.000	0.999	…	…
DAI	1.818 (0.151, 21.962)	0.638	…	…
Posttraumatic LOC	**10.320 (2.177, 48.925)**	**0.003**	**10.545 (1.973, 56.375)**	**0.003**
PTA	**4.286 (1.019, 18.029)**	**0.047**	3.018 (0.530, 17.192)	0.213
Age*	…	0.742	…	…
TI*	…	0.157	…	…
GCS		0.791	…	…
GCS (15)	1		…	…
GCS (14)	1.900 (0.156, 23.135)	0.615	…	…
GCS (13)	1.900 (0.156, 23.135)	0.615	…	…

*Determined with the unpaired student's *t*-test.

Note − SAH indicates subarachnoid hemorrhage; IVH, intraventricular hemorrhage; SDH, subdural hemorrhage; DAI, diffuse axonal injury; LOC, loss of consciousness; PTA, post-traumatic amnesia; TI, time interval; GCS, Glasgow Coma Score.

## Discussion

Our study verified the benefit of contrast-enhanced FLAIR images in detecting traumatic brain injury. In 32 patients, contrast-enhanced FLAIR images clearly demonstrated thick linear enhancement of the dura. Normal dura mater shows only thin, linear, and discontinuous enhancement on contrast enhanced T1-weighted MR images due to lack of sufficient water to generate the T1 shortening required for vivid enhancement. When water accumulates within the dura mater, contrast enhancement can occur [17]. It is well known that various benign or malignant conditions, including intracranial hypotension, granulomatous disease, and metastatic disease can be associated with dural enhancement on contrast-enhanced T1-weighted MR images [17], [18]. In addition, many prior studies have demonstrated that patients who have undergone intracranial surgery show postoperative dural enhancement [19] [19]–[23]. This is held to be due to a local inflammatory response initiated by the bleeding from surgical violation of the dura [21], [1].

Little is known about the relationship between meningeal changes and brain trauma. Sze reported enhancement of the meninges as a result of bleeding into the subarachnoid space after subarachnoid hemorrhage from a presumed aneurysm. The researcher suggested that any process that causes bleeding into the CSF can result in abnormal meningeal enhancement [18]. Similarly, according to pathological-anatomical studies, SDHs are actually intradural hematomas, where the dura comprises the outer layer and the inner layer is formed by the arachnoid, covered by a thin layer of dura border cells [24]–[27]. Early dural enhancement without associated thickening of the meninges is probably caused by vascular congestion and/or increased vascular permeability in the outer dural layer [19], [21].

In our study, 23 out of 28 patients who had any traumatic brain lesions showed meningeal enhancement, while only 5 patients with traumatic brain lesions showed no enhancement. Our results showed association between the presence of SAH or SDH and meningeal enhancement, consistent with the findings of prior studies [6], [19], [21].

According to Mathews et al., contrast-enhanced fast FLAIR images can depict lower concentrations of gadolinium compared with T1-weighted images. The finding that slow-flowing blood is not usually hyperintense on contrast-enhanced fast FLAIR images allows clearer distinction between enhancing meninges or superficial parenchyma and enhancing cortical veins [14]. In our study, the low concentrations of contrast material associated with minor meningeal or venous injury, or with small hemorrhages from trauma, caused prominent meningeal enhancement on contrast-enhanced FLAIR images, as opposed to the equivocal enhancement seen on contrast-enhanced T1-weighted images, which may be confused with cortical veins. The brighter enhancement aided in the detection of additional traumatic brain lesions. In 2 SDHs and 1 SAH, which were initially missed with routine image sequences, prominent meningeal enhancement on contrast-enhanced FLAIR images pointed to small amounts of hemorrhage around the enhancing or opposite convexity on routine image sequences. We expect that the additional finding that the focal convexity meningeal enhancement was usually in a contrecoup location opposite the trauma site may help radiologists to find more lesions.

Interestingly, we found 9 patients without traumatic brain lesions on routine image sequences and only meningeal enhancement along the falx on contrast-enhanced FLAIR images. Traumatic interhemispheric subdural hematoma (ISH) is a known but uncommon complication of head trauma. It is most likely due to laceration of either the parafalcine or parasagittal bridging veins between the medial border of the cerebral hemisphere and the sinus caused by linear brain acceleration during trauma [28], [29]. Although obvious subdural hematoma, which can be demonstrated on routine image sequences, did not arise, we presumed that minor lacerations sufficient for inducing contrast enhancement on contrast-enhanced FLAIR images occurred in the 9 patients. From our results, we found that the incidence of minor injury along the interhemispheric fissure is not rare, as opposed to other reports on ISH.

We found that SDH and posttraumatic LOC were significantly associated with meningeal enhancement. In patients with isolated falcine enhancement, those were still statistically correlated with enhancement. Previous studies have shown poorer neuropsychologic performance after mild TBI complicated by a brain lesion compared with uncomplicated mild TBI despite similar GCS scores between these 2 TBI populations [30]–[32]. Therefore, we believe that the presence of meningeal enhancement implies clinically significant brain injury.

Our study has some limitations. First, in the present study, administration of contrast material is needed. However, many clinicians request a contrast study for various reasons, including evaluation of brain pathology other than traumatic lesion, which may cause the patient's symptoms, or for evaluation of intracranial and extracranial vessels. So, in case of contrast study was performed in patients with mild head injury, our result will be helpful. Moreover, meningeal enhancement depicted on contrast-enhanced FLAIR image can suggest trauma event when there is unclear history of head trauma. Second, the duration of meningeal enhancement was not evaluated, because in a majority of cases, follow-up MR exam was not performed. However, in 3 patients who underwent two MR examinations at 1 day and 1 week after the head injury, 2 patients showed persistent enhancement and 1 patient showed resolution of enhancement on follow-up MR exam. In a patient with 2 follow-up MR studies performed at 1 week and 2 years after the head injury, the meningeal enhancement was decreased after 1 week and resolved after 2 years. We propose that meningeal enhancement becomes less intense over time as a result of resolution of reactive changes related to the presence of blood. However, prior report of persistence of prolonged enhancement and thickening of the dura even years after surgery due to granulation tissue indicate that meningeal enhancement after trauma might be persistent after several years [23]. Third, we could not use SWI as a routine image sequence, since GRE image rather than SWI were routinely obtained in our hospital due to clinician's request during study period. SWI is more sensitive than GRE image for the detection of microhemorrhages, but successful implementation may be difficult due to the longer acquisition time, particularly in head trauma patients who are motion-prone in clinical setting. Moreover, as SWI is more susceptible to artifacts caused by calvaria, susceptibility artifacts arising from field inhomogeneities at the interfaces between bone and tissue, the detection of small SAH and SDH may be limited. Soman et al. reported that GRE was superior to SWI with regard to T2* lesion conspicuity in one case with cortical venous thrombosis due to worsened calvarial artifacts adjacent to the T2* lesion [33].

Despite all these limitations, our study revealed that contrast-enhanced FLAIR images help detect meningeal enhancement in patients with symptomatic closed mild head injuries, and the enhancement often points to traumatic brain lesions. Further research is needed to establish the direct relation between meningeal enhancement on contrast-enhanced FLAIR images and neurocognitive consequences, but the presence of meningeal enhancement on contrast-enhanced FLAIR images may imply significant head injury. Because FLAIR images after administration of contrast material may provide additional information, contrast-enhanced FLAIR MR imaging is recommended when a contrast MR study is indicated in a patient with a symptomatic prior closed mild head injury.
